# Neck Swelling With Valsalva

**DOI:** 10.7759/cureus.42762

**Published:** 2023-07-31

**Authors:** Adibah Azimuddin, Chaithanya Jeganathan, Geoffrey Hee, Peter Friedland

**Affiliations:** 1 Ear, Nose, and Throat Surgery, Sir Charles Gairdner Hospital, Perth, AUS; 2 Otolaryngology, Sir Charles Gairdner Hospital, Perth, AUS

**Keywords:** obstructive sleep apnoea, continuous positive airway pressure (cpap), intrapharyngeal pressure, pharyngeal diverticulum, pharyngocoele

## Abstract

We present a rare case of bilateral pharyngocoeles in a patient with symptomatic neck swelling prominently seen with the Valsalva manoeuvre. Pharyngocoeles have only been reported a handful of times in the literature. Due to their rarity, they can easily be misdiagnosed as a laryngocoele, Zenker’s diverticulum, or jugular venous phlebectasia. The diagnosis in this case was confirmed on computed tomography imaging of the neck with Valsalva performed. Our patient underwent surgical excision of the symptomatic pharyngocoele on the right side while conservative management was opted for the asymptomatic left pharyngocoele. His risk factors for developing bilateral pharyngocoeles are most likely due to the use of continuous positive airway pressure (CPAP) machine at high pressures coupled with pharyngeal wall weakness. To our knowledge, this is the first case of pharyngocoeles associated with CPAP machine use. It is important to perform a thorough assessment to appropriately diagnose and treat patients with this anatomical anomaly.

## Introduction

Pharyngocoele is an outpouching of the lateral pharyngeal wall mucosa [1,2]. First documented in 1886 by Wheeler, they described the symptoms experienced by a military officer [3]. Pharyngocoeles are rare anatomical anomalies of the pharynx and are usually associated with increased intrapharyngeal air pressure, with cases reported in wind instrument musicians, glassblowers, and military personnel [2,3]. It affects more men than women and has a propensity to occur in the fifth to sixth decade of life [1,2,4]. They occur in areas of weakness in the pharyngeal wall, usually affecting the space between the middle and inferior constrictors and the thyrohyoid membrane [2,5]. True lateral pharyngocoeles are rare and can be misdiagnosed; thus, a targeted history, physical examination, and imaging should be performed before treatment. We present a case of a gentleman with a two-year history of unilateral neck swelling.

## Case presentation

A 67-year-old male presented to an ear, nose, and throat clinic with a two-year history of right-sided neck swelling. Interestingly, the swelling increased in size on the Valsalva manoeuvre. He also described the trapping of food fragments resulting in coughing and choking fits. There were no associated dysphagia or voice changes.

He used an auto-set continuous positive airway pressure (CPAP) machine for obstructive sleep apnoea (OSA) with pressures in the range of 10.0-17.5 cmH2O. There was no history of smoking. He was a sheet metal worker and had never played wind instruments or partaken in glass blowing.

**Figure 1:**
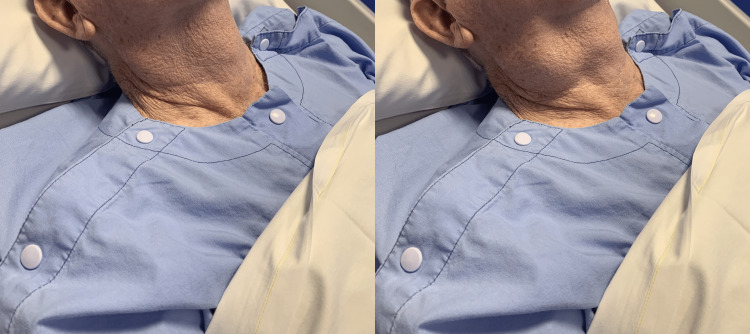
No neck swelling is seen at rest (left). Prominent neck swelling is seen with the Valsalva manoeuvre (right).

On examination, an obvious right neck swelling was seen on Valsalva, which was easily compressible (Figure 1). There was no obvious neck swelling present on the left. A small internal opening in the lateral aspect of the right vallecula was seen on flexible nasoendoscopy. Apart from frothy saliva, there were no other findings on intubation of this opening.

**Figure 2:**
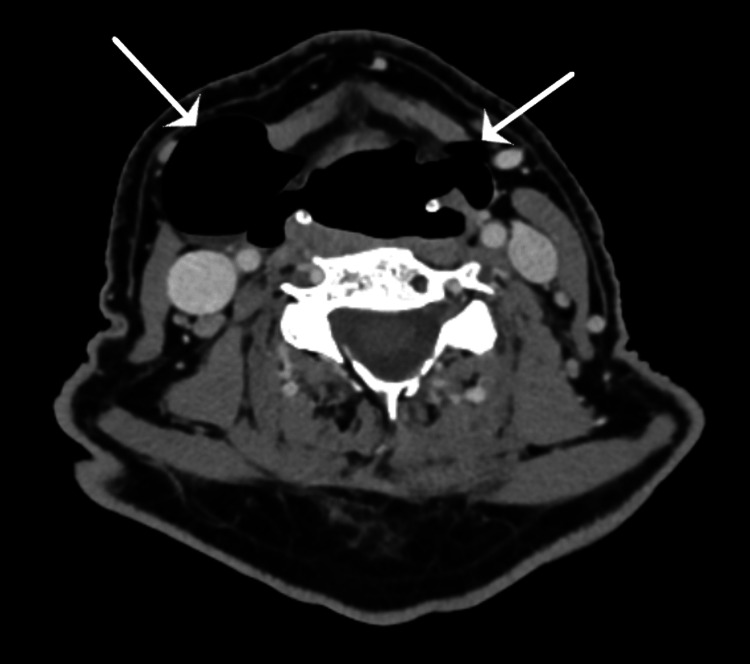
Axial slice of CT of the neck on Valsalva, which shows air-filled sacs inferior to the hyoid penetrating the thyrohyoid membrane more prominent on the right as depicted by the arrows.

A contrast-enhanced computed tomography (CT) imaging of the pharynx with a second acquisition with Valsalva was performed. This imaging was reviewed at a multidisciplinary radiology meeting with head and neck radiologists and otolaryngologists present. Bilateral pharyngocoeles were noted, which distends on Valsalva coming from the valleculae, significantly prominent on the right side (Figure 2). The pyriform fossae also appeared patulous.

The patient was informed of the options, conservative or rather operative management, and informed of the risks of the operation. After discussion, he agreed to proceed with an excision of the right pharyngocoele. The left pharyngocoele was to be managed conservatively as he was asymptomatic on that side.

**Figure 3:**
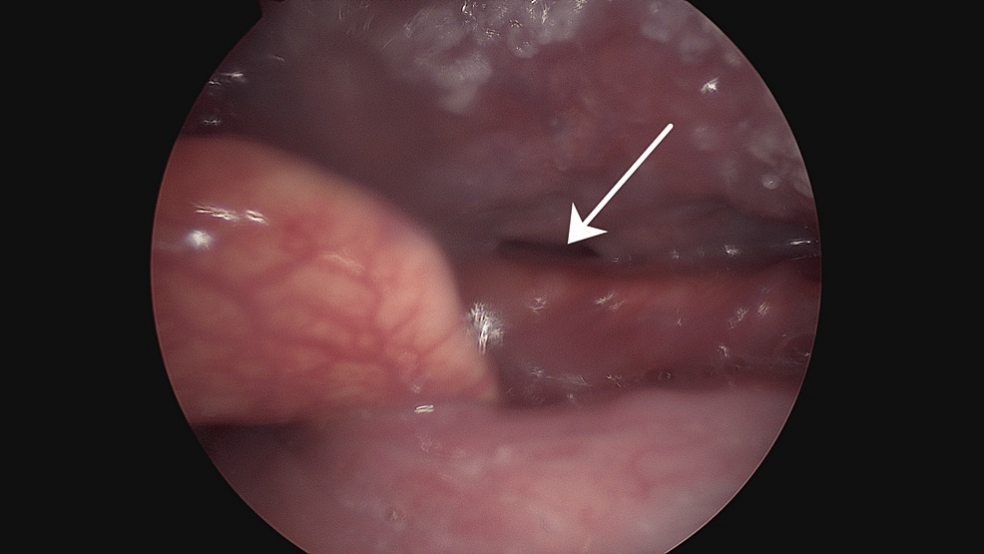
Internal opening in the right vallecula identified intra-operatively.

**Figure 4:**
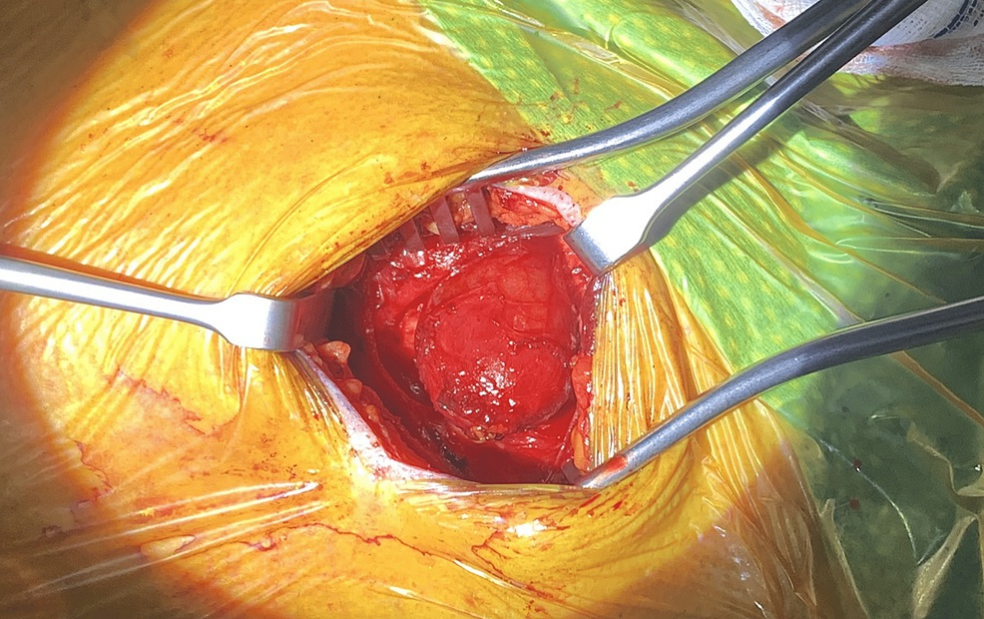
Pharyngocoele identified by packing the internal opening.

On induction of anaesthesia with mask ventilation, the pouch expanded readily at 10 cmH2O. An internal opening was identified in the right vallecula on rigid laryngoscopy (Figure 3). It was then packed with gauze to help identify it from an external approach (Figure 4). A right submandibular skin crease incision was made. The pouch was identified inferior to the right submandibular gland and was bluntly dissected. The pouch was identified inferior to the right submandibular gland and was bluntly dissected. This pouch was then stapler divided. The excised pharyngocoele measured approximately 3.5 cm. A methylene blue test was done to check for leaks. A Blake drain was inserted, and the wound was sutured close. There were no immediate complications post-operatively and the patient was discharged the following day on a soft diet for one week. A CPAP technician was consulted to adjust his CPAP machine to cap the pressure to 10 cmH2O in the peri-operative period.

## Discussion

Only 10 cases of bilateral pharyngocoeles have been reported with only 61 cases of true lateral pharyngocoeles that penetrate the thyrohyoid membrane [4]. They can be either congenital or acquired [2,5]. Congenital cases are unilateral and are often associated with a branchial cleft remnant [2,5]. Acquired cases are more frequent and are thought to be due to pharyngeal wall weakness associated with age, increased intrapharyngeal pressures, and previous surgeries [2,4,5]. There are two areas of weakness within the pharyngeal wall, which are the regions between the superior and middle constrictors and between the middle and inferior constrictors [1,2]. As a result of ageing and increased intrapharyngeal pressures, these areas can form protrusions of pharyngeal mucosa forming pharyngocoeles [1,2]. The literature mainly describes cases of glassblowers and wind instrument musicians [2,4,5]. Patients with symptomatic pharyngocoeles typically present with dysphagia, dysphonia, regurgitation, cough, or odynophagia [1].

Pharyngocoeles can be misdiagnosed as laryngocoele, Zenker’s diverticulum, and jugular venous phlebectasia [4,6]. Imaging can be used to distinguish pharyngocoeles from other differentials. The most common imaging modalities used include barium swallow and CT imaging with Valsalva. As described by Xiang et al., a “bullfrog” appearance is seen on frontal cervical X-rays or barium swallow in bilateral pharyngocoeles when performed with Valsalva, which outlines the pharyngeal outpouching [7]. On CT, an outpouching of the pharyngeal wall through the thyrohyoid membrane is seen. It becomes more pronounced on the Valsalva manoeuvre whereby an air-filled sac can be identified expanding the pyriform sinus through the thyrohyoid membrane [5]. Traser et al. describe the use of dynamic real-time and three-dimensional MRI that examines pharyngocoeles with speech and swallowing [8].

Our patient presented with the symptoms associated with a pharyngocoele, which was confirmed on CT imaging. His main risk factor was most likely the use of a CPAP machine, leading to an increase in intrapharyngeal pressure. According to Gold et al., the critical pharyngeal pressure will need to be exceeded with CPAP to prevent pharyngeal collapsibility by forming a “splint”, which in turn increases intrapharyngeal pressure [9]. High intrapharyngeal pressures coupled with congenital or age-related muscle weakness may have precipitated the development of bilateral pharyngocoeles in this case. To our knowledge, this is the first report of pharyngocoeles associated with CPAP use in the literature.

The treatment of pharyngocoeles has been varied and usually depends on the severity of symptoms. In patients who are largely asymptomatic or where the discomfort is only mild, conservative management is the mainstay. Neck trusses or bands can be used to assist with preventing outpouching. Surgical management of pharyngocoeles is generally performed with an external incision [4,5,10].

## Conclusions

Pharyngocoeles are uncommon and are most likely acquired from an increase in intrapharyngeal pressures and muscle weakness. The most probable aetiology in this case is the use of CPAP, which resulted in high intrapharyngeal pressures aggravating pharyngeal wall weakness associated with ageing. As they may present similar to a number of other diagnoses, they tend to be misdiagnosed. It is important to acquire an accurate history, perform a thorough examination, and obtain appropriate imaging to correctly diagnose this anomaly. Pharyngocoeles can be managed both conservatively and surgically. Surgical management should be considered in symptomatic patients.
